# Sensitivity of a Subject-specific Ankle Sprain Simulation to Extrinsic Versus Intrinsic Biomechanical Factors

**DOI:** 10.3389/fbioe.2021.765331

**Published:** 2021-12-08

**Authors:** Adam J. Yoder, Anthony J. Petrella, Shawn Farrokhi

**Affiliations:** ^1^ DoD-VA Extremity Trauma and Amputation Center of Excellence, San Diego, CA, United States; ^2^ Department of Physical and Occupational Therapy, Naval Medical Center, San Diego, CA, United States; ^3^ Department of Mechanical Engineering, Colorado School of Mines, Golden, CO, United States; ^4^ Uniformed Services University of the Health Sciences, Bethesda, MD, United States

**Keywords:** ankle, inversion, injury, sprain, instability, brace, opensim

## Abstract

Ankle sprains are the most common musculoskeletal injury in sport and military activity, despite existing prophylactic strategies. The purpose of this report was to develop a probabilistic simulation of lateral ankle sprains during single-limb drop landing, towards accelerating innovation in ankle sprain prevention. A deterministic, subject-specific musculoskeletal model was extended with automation and probabilistic distributions on sprain-related biomechanical factors. Probabilistic simulations were generated using traditional Monte Carlo techniques and the advanced mean value method, a more computationally-efficient approach. Predicted distributions of peak ankle joint rotations, velocities, and moments borne by supporting passive structures agreed favorably with the deterministic model and with reports of real sprain biomechanics. Parameter sensitivities identified that predictions were most strongly influenced by drop height, subtalar joint posture at contact, invertor/evertor co-activation, and passive ankle stiffness. The advanced mean value method predicted confidence bounds comparable to a 1000-trial Monte Carlo simulation, and required only 14 model evaluations and 4-min processing time. The extended probabilistic simulation may be useful to virtually test new prophylactic strategies for ankle sprains, and is made available for open-source use (https://simtk.org/projects/sprain-sim).

## Introduction

Ankle sprains are the most common musculoskeletal injury in sport and military physical activities requiring medical care, with estimated prevalence as high as 10–12% (Ruscio et al., 2010; Doherty et al., 2014). Among individuals who experience a first-time sprain, 40% will go on to develop chronic ankle instability, which can lead to recurrent sprains, time lost at work, and decreased quality of life (Doherty et al., 2016). Better preventative interventions are needed to reduce the burden of sprains on individuals and healthcare systems.

The most strongly recommended intervention to date for prevention of first-time or recurrent lateral ankle sprains is use of a semi-rigid brace during high-intensity physical activity (Newman et al., 2017; Martin et al., 2021). However, negative perceptions of ankle braces among users and prescribing clinicians exist, which influence use and abandonment, such as: interference with mobility, low satisfaction with comfort, and risks of dependency or decreased muscle strength over time (Gross and Liu, 2003; Denton et al., 2015; Dierker et al., 2017).

A framework to directly study sprain mechanics while rapidly testing different prophylactic strategies across varied users and movements could accelerate innovation. Direct measurement of sprain mechanics with injury potential is unethical, and thus sub-injury experiments on human subjects (e.g., “inversion platforms” (Ha et al., 2015)), or laboratory study on post-mortem models, have been the standard. However, such approaches do not fully represent sprain-causing motion and loads, or are time and cost intensive even with small sample sizes.

Computational simulation of musculoskeletal injury can supplement experiments to bridge these gaps (Seth et al., 2018). Forward dynamic, muscle-driven simulation has been used to isolate relative influences of foot positioning (Wright et al., 2000b), passive ankle flexibility (Wright et al., 2000a), and invertor/evertor muscular control on ankle sprain occurrence (DeMers et al., 2017). Modeling assumptions are often a primary limitation, although expected uncertainties can be incorporated with probabilistic techniques to increase confidence (Myers et al., 2015). Probabilistic simulations have been used to inform design of orthopaedic joint implants (Easley et al., 2007), quantify the influence of ankle-foot orthosis design on muscle mechanics in children with cerebral palsy (Hegarty et al., 2017), and to evaluate prophylactic roles of neuromuscular control versus movement strategy in sport knee injury (McLean et al., 2008). An efficient, probabilistic musculoskeletal simulation framework to study ankle sprain biomechanics that incorporates both intrinsic human factors and extrinsic factors has yet to be tested.

The primary purpose of this study was to extend a deterministic, subject-specific ankle sprain model for efficient, probabilistic simulation. Parameter sensitivities were characterized, and probabilistic predictions were validated against the original model and reports of real sprain occurrences.

## Methods

### Musculoskeletal Model

**FIGURE 1:**
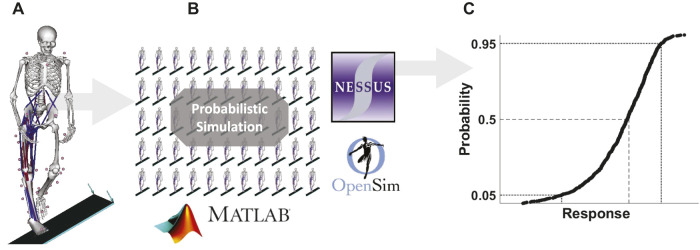
**(A)** Baseline deterministic musculoskeletal model landing on a 30° incline (DeMers et al., 2017). **(B,C)** The model was extended with OpenSim API automation (version 3.3) and NESSUS software (v9.8.0, SwRI, San Antonio, TX) for probabilistic simulation.

A publicly-available musculoskeletal model was expanded upon for this study (DeMers et al., 2017) (Figures 1A). Briefly, the whole-body model (49 muscles, 21 degree-of-freedom) contained elastic foundation foot-ground contact (Seth et al., 2018) and lumped passive ankle stiffness represented by an uncoupled, rotation-only bushing (Chen et al., 1988). Joint motion was driven by custom stretch-reflex feedback muscle controllers. For baseline validation, the generic model anthropometry was scaled to a single human subject (female, 68 kg, 180 cm), and muscle control parameters optimized to track whole-body joint kinematics measured via 3D motion capture during a 40 cm drop onto a level surface. The model also had a landing platform that could be inclined to induce virtual inversion sprains. The unmodified, subject-specific model was used as the mean baseline model for probabilistic simulations.

**FIGURE 2:**
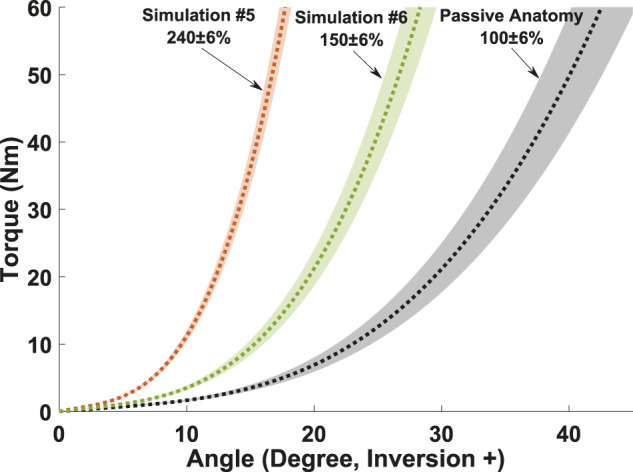
Passive bushings were used to separately model the net effect of passive anatomy (ligaments, cartilage, etc.) and protective bracing applied between tibia and calcaneus reference frames. Inversion angle corresponds to rotation about an anterior-posterior, calcaneus-fixed axis. Applied bracing was scaled as a function of passive anatomy to aid physical interpretation. Angular displacement values at fixed torque levels were multiplied, such that a brace with a 150% scale factor would yield 50% less excursion, relative to baseline passive anatomy under the same applied torque. Flexibility was scaled, versus compliance (multiplying torque data), as flexibility has been shown more influential in prior ankle sprain simulations (Wright et al., 2000a).

We also extended the baseline model with a representation of external, ankle-foot structural support (e.g., a brace, Figure 2). The rotation-only bushing representing passive anatomy was first duplicated, then scaled to represent adjustable magnitudes of bracing acting in parallel with passive anatomy. Specifically, angular displacement data was multiplied with a fixed factor (i.e., scaling flexibility (Wright et al., 2000a)), such that a brace with a 150% multiplier would be 50% less flexible than the model’s passive anatomy. This approach was chosen as ankle flexibility versus compliance (scaled torque at fixed displacements) –has been suggested to be more influential in sprain prevention (Wright et al., 2000a).

### Probabilistic Simulation

After verifying reproducibility of the baseline deterministic simulation (DeMers et al., 2017), we assigned probabilistic distributions to 10 input parameters expected to influence sprain occurrence (Table 1). Mean values were kept equal to the baseline model, while assumed variances were relatively minor perturbations aimed to preserve validity of the baseline model (Table 1). Monte Carlo (MC) simulations with 1000 trials were instantiated with random sampling (NESSUS, v9.8.0, SwRI, San Antonio, TX) and executed via the MATLAB-OpenSim API (version 3.3). Six separate probabilistic simulations were generated with a level or 30-degree inclined landing surface; each using a common set of 7 parameter distributions and a combination of muscular co-activation, reflexive gain, and external bushing stiffness (Table 1).

**TABLE 1:** Probabilistic input parameters to six Monte Carlo simulations of single-limb drop landing. Each simulation was generated using 10 total parameters, where each had a fixed landing surface incline, a common set of 7 probabilistic parameters, and either fixed zero (0) or variable ranges of muscular co-activation, reflex gain, and brace flexibility.

Input Parameter	Distribution	Mean ± SD					
Monte Carlo Study #		1	2	3	4	5	6
Landing surface incline (^º^, degrees)	fixed	0	30	30	30	30	30
Brace Flexibility (% of passive anatomy flexibility)	normal	0	0	0	0	240 ± 6	150 ± 6
Muscle co-activation (invertor/evertor, %)	lognormal	0	0	0	60 ± 5	0	20 ± 5
Muscle reflex gain (invertors, evertors)	lognormal	0	0	10 ± 1	0	0	5 ± 1
Muscle strength (invertor/evertor, % baseline)	normal	100 ± 5					
Ankle passive flexibility (% baseline)	normal	100 ± 6					
Joint angle, talocrural at contact (^º^)	normal	34 ± 5					
Joint angle, subtalar at contact (^º^)	normal	0 ± 5					
Drop height (meters)	lognormal	0.30 ± 0.05					
Ground-foot contact modulus (MPa\meter)	lognormal	50 ± 5					
Ground-foot contact dissipation (sec\m)	lognormal	5 ± 1					

### Data Analysis

**FIGURE 3:**
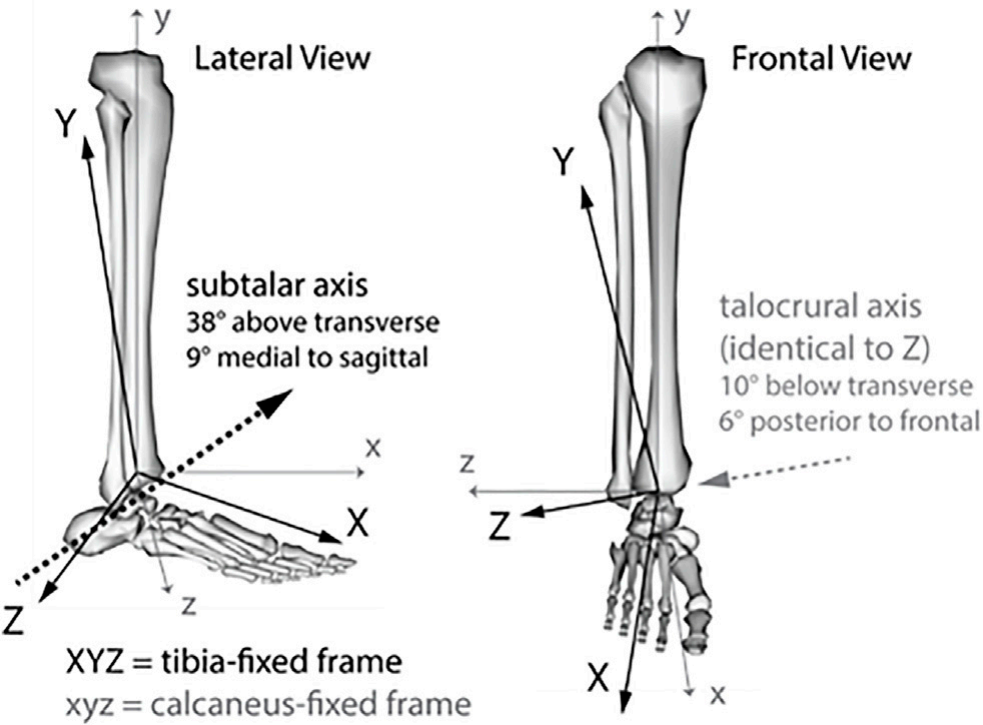
Orientation of internal anatomic axes for the subtalar and talocrural joints, as defined in the generic, baseline musculoskeletal model (Inman, 1976; Delp et al., 1990).

Rotational kinematics of the ankle were kept as defined in the baseline, generic OpenSim model. Briefly the model contained two body-fixed, anatomic axes (Inman, 1976; Delp et al., 1990). Talocrural motion (dorsiflexion (+)/plantarflexion) was defined as rotation of the talus around an oblique, talocrural axis fixed in the tibia (Figure 3). Subtalar motion (supination (+)/pronation) was defined as rotation of the calcaneus around an oblique, subtalar axis fixed in the talus (Inman, 1976). Kinematics were additionally resolved post-hoc to a joint coordinate system (JCS) for the combined ankle complex recommend by the International Society of Biomechanics (ISB) (Wu et al., 2002), which measures non-orthogonal rotations of the body-fixed calcaneus frame relative to the body-fixed tibia frame. The JCS sequence was per convention: dorsiflexion (+)/plantarflexion about a tibia-fixed Z-axis, which was identical to the talocrural anatomic axis; inversion (+)/eversion rotation around a floating axis; and internal (+)/external rotation around a body-fixed calcaneus *y*-axis (Grood and Suntay, 1983; Wu et al., 2002).

Peak subtalar supination angle was extracted as the primary outcome from probabilistic analysis, along with several complementary biomechanical metrics to aid interpretation and validation: peak talocrural angle, peak angular velocities, and moments borne by each passive bushing at the instant of peak supination. Bushing moments, expressed as vectors in the global reference frame by default, were orthogonally projected (i.e., dot products) onto each anatomic and JCS coordinate axis.

Cumulative distribution functions (CDF) of peak subtalar supination angle were computed from each MC simulation. The 5th, 50th, and 95th percentile values were extracted for comparison (Figures 1C). Sensitivity of the response to each independent parameter was characterized with standard Pearson correlation coefficients, and probabilistic sensitivity factors—which offer a unique assessment of how the mean and variance of the response are influenced by changes in either the mean or variance of each input (Easley et al., 2007).

### Advanced Mean Value Method

We also tested the accuracy of the advanced mean value (AMV) probabilistic analysis method, a more computationally-efficient approach relative to traditional MC methods (Wu et al., 1990). Briefly, the AMV method approximates the numerical model (i.e., the sprain simulation) as a first-order Taylor series expansion to estimate the response gradient at specified probability levels. The AMV method requires only tens of trials to generate results, but can be sensitive to non-monotonic system behavior. Thus, we used our MC results as a gold standard to test if AMV predictions were similar at 5, 50, and 95% performance levels using the same inputs. Lastly, we evaluated AMV importance levels, which are the components of a unit vector pointing in the direction of the system response gradient. Thus, importance levels at each performance level have a root-mean-square sum of unity, and provide a more concise sensitivity analysis.

## Results

**FIGURE 4:**
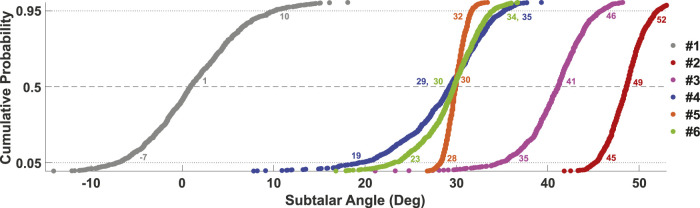
Subtalar joint peak angle (supination, +) in response to varied intrinsic and extrinsic sprain parameters across six Monte Carlo simulations (Study #1-#6, Table 1). Response values corresponding to the 5^th^, 50th, and 95th percentile were extracted from cumulative distributions for comparison (horizontal dotted lines).

Cumulative distributions for the 6 MC studies are illustrated in Figure 4. Study #1, which simulated drop landing on a level surface, predicted the lowest values of peak supination angle among all studies but also had the largest uncertainty in response with a range of 17° between 5 and 95% bounds, driven by subtalar posture at landing (Table 2). Study #2 confirmed that with addition of a 30-degree incline, and still without brace protection or invertor/evertor muscle activity, the most severe peak supination angles were predicted, with a median response of 49° and 90% of trials falling in the range 45-52°. Response was decreased most strongly driven by passive anatomic stiffness, and moderately by subtalar supination posture at landing (Table 2).

**TABLE 2:** Correlations between probabilistic input parameters and peak subtalar supination angle within six Monte Carlo simulations. Input parameter distributions are provided in (Table 1). Probabilistic sensitivity factors were additionally computed to assess independent influences of input parameter mean and variance on response mean and variance, and generally agreed with correlations, with exception of ( $\sigma_{-}^{+}$ ) indicating an increase in the parameter mean increased (+) or decreased (-) variance in the response. Supplementary Figure S6, S7).

Monte Carlo Study (#)	1	2	3	4	5	6
Drop height	0.10	0.36^a^	0.61^c^	0.34^a^	0.65^c^	0.61^c^
Ground contact compliance	−0.01	0.19	0.22	0.16	0.20	0.12
Ground contact dissipation	−0.01	-0.38^a^	−0.07 ( $\sigma_{-})$	0.01 $\left(\sigma_{-}\right)$	0.00	0.02
Muscle co-activation	─	─	─	−0.37^a^	─	−0.36^a^
Muscle reflex gain	─	─	−0.05	─	─	−0.01
Muscle strength, inv/evertor	0.03	−0.08	−0.08	−0.21^a^	−0.05	−0.16
Talocrural joint plantarflexion at contact	−0.10	0.18	0.28 $\left(\sigma^{+}\right)$ ^a^	−0.02 $\left(\sigma^{+}\right)$	−0.50^b^	0.16
Subtalar joint supination at contact	0.99^c^	-0.42^b^	0.55^b^	0.74^c^	0.10	0.57^b^
Passive anatomic stiffness	−0.06	−0.67^c^	−0.15	−0.07	−0.08	−0.03
External brace stiffness	─	─	─	─	−0.49^b^	−0.13

Correlation strength: weak, (*r* = 0.2–0.4)^a^, moderate (*r* = 0.4–0.6)^b^, strong (*r* = 0.6–1.0)^c^.

( $\sigma_{-}^{+}$ ) identifies a probabilistic sensitivity factor that suggested increased input parameter MEAN, value led to increased (+) or decreased (-) variance in the response.

Study #3 which added invertor/evertor muscular reflexes verified that strong reflexes only marginally reduced peak supination to 41° at the 50% response level. Sensitivities showed that despite presence of reflexes, response was most strongly increased by drop height, and moderately increased by subtalar supination posture at landing (Table 2). Study #4 which excluded muscular reflexes and added preparatory co-activation, predicted a large reduction in peak subtalar response to 30°, but also showed a relatively wide 90% confidence bound, ranging 16°. Response was most strongly increased by subtalar joint posture at landing and a weak/moderate negative association with co-activation (Table 2).

For both Study #3 and #4, probabilistic sensitivity factors highlighted that assumed increases in some input parameters influenced variance in peak subtalar response; specifically, increased mean ground contact dissipation decreased response variance, and increased mean talocrural joint posture at landing increased response variance (Table 2).

Study #5 and #6 which each incorporated added external structural support (i.e., a brace), showed similar reductions in peak subtalar response at 30° (Figure 4). However, Study #5 had tighter confidence bounds, with moderate, negative subtalar response sensitivity to talocrural angle at contact and to brace stiffness. In contrast, Study #6 that included a mix of all considered factors, showed response was primarily predicted by an increased subtalar angle at contact, and drop height (Table 2).

**FIGURE 5:**
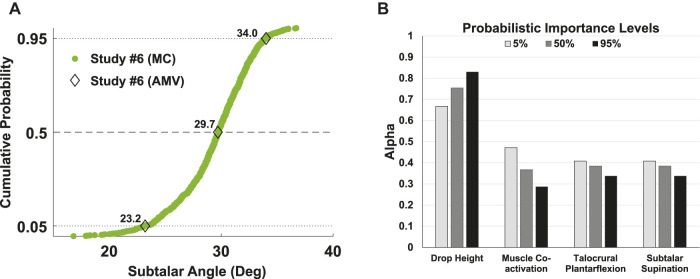
**(A)** Advanced Mean Value (AMV) probabilistic method in comparison to traditional Monte Carlo (MC) for Study #6 (Table 1). For 10 input parameters, the efficient AMV method required only 14 model evaluations to predict response probabilities (0, 5, 50, 95%) which agreed with a 1000-trial MC. The AMV method executed in 4 min processing time versus 13 hours for MC (laptop, Intel i7 CPU). **(B)** Probabilistic Importance Levels computed using the AMV method. Alpha values at each probability level are the components of a unit vector pointing in the direction of the response gradient (steepest ascent). The root-mean-square sum of alpha values is unity and thus establish a relative ranking of input parameters to which the response is most sensitive. For exemplary Study #6, only four of 10 input parameters had non-zero alpha values which suggested that drop height was the most influential, and showed increasing influence between 5 and 95% response levels, followed by talocrural/subtalar joint postures and muscle co-activation, the latter of which showed decreasing influence between 5 and 95% response levels.

Lastly, the AMV method was successfully implemented by re-executing Study #6 (Figure 5). Only 14 simulation trials and 4-min processing time were required to reproduce 5, 50, and 95% response levels predicted with a 1000-trial MC study accurately and much more efficiently.

## Discussion

The primary aim of this study was to develop a probabilistic simulation of ankle inversion sprains for virtual study of prophylactic interventions. This was achieved by extending a validated deterministic model with several features. Firstly, a comprehensive set of 10 deterministic parameters were assigned probabilistic distributions such that confidence bounds on outputs were predicted, and underlying probabilistic sensitivities characterized. Secondly, an adjustable ankle-foot bushing was added to facilitate exploration of applied mechanical support (i.e., a brace) in parallel with anatomy. Lastly, code was included to report ankle biomechanics using the ISB-recommended joint coordinate system for the ankle complex, which will facilitate inter-study comparison. The extended probabilistic framework is made available for community use (https://simtk.org/projects/sprain-sim).

A breadth of prior human subject experiments report kinematics, kinetics, and muscle activity of the ankle complex during unilateral landing (Ha et al., 2015; Simpson et al., 2019). These observational studies describe cross-sectional differences between groups with and without ankle instability, characterized by altered neuromuscular control and passive joint laxity (Simpson et al., 2019). However, sub-sprain experiments do not fully model injurious motion and loads, nor can load sharing among structural elements (e.g., muscles, ligaments, bracing) be easily determined. Computational sprain simulations have been used to explore underlying factors in sprain occurrence; specifically, the influence of foot posture at landing (Wright et al., 2000b), passive ankle flexibility (Wright et al., 2000a), and muscular co-activation versus reflex strength (DeMers et al., 2017). However, no single framework has incorporated a breadth of human factors in combination with external factors and understood uncertainties. Additionally, a vast majority of biomechanical studies resolve ankle-foot mechanics using the ISB JCS (Wu et al., 2002), the axes of which differ from ankle internal anatomic axes, such as those implemented in OpenSim. Thus, our extended framework resolves ankle-foot mechanics in both anatomic and JCS conventions to facilitate comparison across studies.

### Comparison With Baseline Deterministic Model

The original purpose of the validated deterministic model was to explore if muscular co-activation or reflexes could theoretically prevent a sprain (DeMers et al., 2017). Through uniform variation of preparatory invertor/evertor co-activation and reflex gain (20 total simulations), the authors of the prior study concluded that co-activation could prevent sprains, while reflexes could not; specifically, 60% co-activation reduced peak subtalar supination from 49 to 30°, while the strongest reflexes (gain of 10) produced an unsafe 41° supination. Our corresponding, probabilistic simulations - Study #3 with strong reflexes (gain 10 ± 1), Study #4 with strong co-activation (60 ± 5%) - aimed to verify if these conclusions held with added uncertainty across a breadth of sprain factors (Table 1). Median response of each MC simulation matched deterministic predictions, although 90th percentile ranges were relatively wide (16°, Study #4, Figure 4). With co-activation only, our sensitivity results showed response variance was most strongly driven by subtalar posture at contact (*r* = 0.74, Study #4, Table 2), with only weak associations across co-activation, drop height, and muscle strength (*r* = −0.37, 0.34, −0.21). With reflexes only, 95% of our trials produced 35° or greater peak supination (Study #3, Figure 4), with response variance showing strong associations to drop height and subtalar posture at contact (*r* = 0.61, 0.55, Study #3, Table 2), but not to reflex gain. These findings corroborate prior conclusions that—even with added intrinsic and extrinsic uncertainty—the strongest reflexes could not prevent sprains for this specific subject and movement (DeMers et al., 2017).

### Ankle Joint Mechanics

Our analysis of predicted ankle joint mechanics expressed in a JCS highlighted several points. Firstly, 35 ± 6° inversion has been suggested as a range within which lateral ligament damage beings to occur in cadaveric models (with foot 20° plantarflexed, 15° internally rotated), measured about an anterior-posterior axis parallel to the plane of the foot, through the talocrural joint (Aydogan et al., 2006). In this simulation, the generic subtalar joint axis is inclined 38° superior and 9° medial to anatomic directions of the foot (Figure 3). Thus, with 20° talocrural plantarflexion, 52° subtalar supination is required to yield 35° inversion in JCS coordinates, highlighting care needed when comparing across different conventions. As the ISB ankle complex JCS is more commonly used in human motion capture experiments, resolution of default OpenSim outputs to a JCS facilitates comparison. For example, one experiment had subjects drop land onto a 25° inclined platform, and captured 3D kinematics and kinetics of the JCS ankle complex during safe versus incidental sprain-causing trials in two female participants (Li et al., 2018). A range of 35–43° peak inversion in a JCS convention was measured during safe trials, and 55° during sprain trials. Our probabilistic simulations of a 30 cm drop onto a 30° incline predicted 11°—23° peak JCS inversion (Table 3); notably lower than observed injury ranges. Similarly, 600–900°/second inversion velocities were measured during sprain trials, while our simulations predicted a lower 500–700°/second (Li et al., 2018). More severe, virtual sprains could be achieved with a steeper platform incline, or higher drop height, in future applications requiring such.

**TABLE 3:** Predicted, peak ankle mechanics (mean ± standard deviation) across six, 1000-trial Monte Carlo simulations. Input parameter combinations are specified in (Table 1). Kinematics corresponding to subtalar and talocrural axes were extracted directly from the OpenSim model (Inman, 1976; Delp et al., 1990). Kinematics for a non-orthogonal joint coordinate system (JCS) of the tibia-calcaneus ankle complex were also resolved (Figure 3) (Wu et al., 2002). Peak moment borne by each passive bushing (anatomy, *brace* ^b^) at the instant of peak supination were computed as orthogonal projections of ground-referenced moments onto each anatomic and JCS axis.

Monte Carlo Study (#)			1	2	3	4	5	6
		Angle	1 ± 5	49 ± 2	41 ± 3	29 ± 5	30 ± 1	29 ± 3
Subtalar Axis (Sup. +)		Velocity	135 ± 82	886 ± 72	878 ± 82	671 ± 115	911 ± 208	799 ± 81
		Moment	0.7 ± 1.1	31.0 ± 6.1	15.1 ± 4.9	4.3 ± 1.9	4.9 ± 0.8 *61.8 ± 5.0* ^b^	4.3 ± 1.6 *13.2 ± 5.0* ^b^
		Angle	17 ± 1	19 ± 2	19 ± 1	18 ± 2	21 ± 1	20 ± 2
Talocrural Axis (Dorsi. +)		Velocity	1650 ± 365	1684 ± 356	1644 ± 349	1568 ± 337	2284 ± 717	1675 ± 365
		Moment	4.4 ± 0.7	-5.6 ± 1.8	-3.0 ± 1.0	0.9 ± 1.0	2.5 ± 0.5 *11.5 ± 3.5* ^b^	1.5 ± 0.5 *2.2 ± 1.2* ^b^
Ankle-Foot Complex JCS	Sagittal (Dorsi. +)	Angle	19 ± 1	8 ± 1	8 ± 1	10 ± 2	13 ± 1	11 ± 1
		Velocity	1622 ± 347	1641 ± 330	1604 ± 326	1532 ± 315	2324 ± 743	1641 ± 348
		Moment	4.4 ± 0.7	1.0 ± 0.5	0.9 ± 0.7	1.2 ± 0.9	2.6 ± 0.5 *12.4 ± 3.4* ^b^	1.6 ± 0.4 *2.4 ± 1.1* ^b^
	Frontal (Inv. +)	Angle	−9 ± 4	23 ± 1	19 ± 2	11 ± 3	12 ± 1	11 ± 2
		Velocity	103 ± 62	650 ± 57	652 ± 60	501 ± 88	702 ± 167	599 ± 62
		Moment	0.3 ± 0.9	24.5 ± 4.6	13.2 ± 3.8	4.8 ± 1.6	5.3 ± 0.8 *59.5 ± 4.4* ^b^	4.8 ± 1.4 *13.5 ± 4.1* ^b^
	Transverse (Int. +)	Angle	7 ± 3	39 ± 2	33 ± 3	24 ± 3	25 ± 1	24 ± 2
		Velocity	77 ± 48	572 ± 58	537 ± 55	398 ± 68	520 ± 117	471 ± 48
		Moment	0.3 ± 0.5	10 ± 1.6	5.5 ± 1.6	1.9 ± 0.9	2.0 ± 0.4 *23.9 ± 3.1* ^b^	2.0 ± 0.7 *6.1 ± 2.1* ^b^

Selected kinetics were also resolved to the non-orthogonal ankle complex JCS (Table 3). Simulated inversion torque resisted by the rotational bushing representing internal passive anatomy, peaked at 24.5 ± 4.6 Nm in Study #2 in the worst-case of no invertor/evertor muscular contributions or external bracing, and decreased to 4.8–5.3 Nm on average in MC Study’s #4-6 aimed to simulate near-injury scenarios. These latter predictions agree with measurements from the same cadaveric test data (Aydogan et al., 2006); which observed anterior talofibular ligament damage initiation at 2.7–4.3 Nm external inversion torque and 35 ± 6° inversion.

### Effect of Ankle Complex External Mechanical Support

Our addition of a second passive, rotational bushing was aimed to provide a simple means of estimating load sharing between internal anatomy and the net structural effect of a worn brace. Such human-device interactions are difficult or impossible to measure experimentally, but could be useful to inform mechanical design requirements for bracing. Torque-angle data used in the generic bushing definitions can be safely measured on humans with/without worn braces, to provide subject-specific inputs of passive flexibility and external bracing (Siegler et al., 1997; Crabtree and Higginson, 2009). For example, MC Study #5 suggested that - in the absence of invertor/evertor muscular contributions - a parallel, external bushing with a 240% scale factor (Figure 2) would be needed to achieve a similar supination reduction as with 60 ± 5% muscular co-activation alone (Study #4 Table 3). However, the complete absence of muscular contributions is unlikely, and such a rigid brace would likely be uncomfortable during typical ankle motions. Thus, we tested a more realistic scenario including low co-activation, moderate reflex gain, and applied bracing in combination (Study #6, Figure 4 and Table 3). Predictions suggested a more flexible 150% scaled bushing could provide similar protective effect (Figure 4). Future applications may use this framework to estimate design requirements on magnitudes of structural ankle support needed for sprain prevention, to achieve an effective but minimally-restrictive brace design (Dierker et al., 2017). Conversely, structural flexibility of a prototype brace and of a user’s ankle complex can be measured (Siegler et al., 1997), and input to this framework to safely predict probability of a sprain.

### Probabilistic Simulation

An additional aim of this study was to characterize model sensitivities. Standard input-response Pearson correlations generally agreed with probabilistic sensitivity factors computed from MC results (Table 2); moderate and strong correlations corresponded with probabilistic sensitivities that suggested input parameter means drove peak subtalar supination. Increasing variance in correlated parameters also led to increased variance in the response. However, a unique feature of probabilistic sensitivity factors is identification of input-output relationships beyond linear correlation. For example, in MC studies #3 and #4, increased mean ground contact dissipation decreased variance in the response. Similarly, increased plantarflexion at contact increased variance in the response. These relationships are physically sensible and increase confidence in model validity. Specifically, in the absence of external bracing, increased foot-ground damping created a less variable subtalar response, while greater plantarflexion has potential to increase response variance through variance in location of foot-ground contact and moment arms of primary evertors (e.g., peroneus, extensor digitorum). Of note, invertor/evertor muscle strength showed no association with peak subtalar supination across all scenarios. This agrees with clinical studies that have found no predictive relationship between evertor weakness and lateral ankle sprain occurrence (de Noronha et al., 2006; Martin et al., 2021).

Lastly, while probabilistic analysis is a powerful supplement to forward dynamic musculoskeletal simulation, it is often not feasible due to time constraints of traditional techniques. For this reason, we tested the efficient AMV method on Study #6, which included all 10 probabilistic inputs, and found AMV accurately replicated MC predictions at the 5, 50, and 95% levels (Figures 5A). Only 14 simulations were required for the AMV analysis (4 min processing time, laptop Intel i7 CPU), in contrast to the high computational cost of MC (1000 trials, 13 h). Another added benefit of the AMV method relative to MC are probabilistic importance levels, which provide a more succinct, relative ranking of sensitivities at each probability level. For example, importance levels for Study #6 revealed that drop height was the most influential, with increasing effect between 5 and 95% response probability, followed by talocrural and subtalar joint postures, and muscle co-activation, the latter of which showed decreasing influence between 5 and 95% probability levels (Figures 5B).

### Limitations and Future Work

The probabilistic framework and study findings must be considered with several limitations. The experimental data used to validate the baseline model was limited to one subject (female, 68 kg, 180 cm), and one motion (40 cm unilateral drop landing onto a level surface). Therefore, conclusions should not be generalized to substantially different contexts of use without further validation (Viceconti et al., 2020). Comparison to experimental data of persons with varied anthropometry, landing on inclined surfaces, with/without ankle instability could broaden future utility. An added challenge is the definition of injury thresholds to internal anatomic structures via externally-measured joint kinematics or kinetics. Therefore, we chose not to assume a fixed injury threshold, as the primary focus of this study was on incorporating appropriate input uncertainties and characterizing the most influential sensitivities.

## Conclusion

This study successfully developed an efficient probabilistic, subject-specific simulation of ankle inversion sprains during drop landing. This was accomplished through extensions to an open-source, validated deterministic model with probabilistic inputs, characterizing sensitivities, and evaluating confidence bounds on extended predictions. Additionally, ankle joint mechanics were resolved in both default anatomic joint conventions, and in ISB-recommended conventions to facilitate interstudy comparisons (Wu et al., 2002). Lastly, a method was added to estimate load sharing between internal anatomy versus external structural ankle support (e.g. ligaments versus bracing), aimed to accelerate virtual testing of future brace concepts. The extended model and all associated code are made available for open-source use (https://simtk.org/projects/sprain-sim).

## Data Availability

Publicly available datasets were analyzed in this study. This data can be found here: https://simtk.org/projects/sprain-sim.

## Author Disclaimer

The views expressed herein are those of the author(s) and do not necessarily reflect the official policy or position of the U.S. Department of Defense, Department of Energy, ORISE, or the United States Government. Inclusion of brand and product names does not constitute endorsement. This work was prepared as part of official duties of United States Government employees. Title 17, U.S.C., §105 provides that copyright protection under this title is not available for any work of the U.S. Government. Title 17, U.S.C., §101 defines a U.S. Government work as a work prepared by a military Service member or employee of the U.S. Government as part of that person’s official duties.
