# Supplementary material for: Sensitivity of a Subject-specific Ankle Sprain Simulation to Extrinsic Versus Intrinsic Biomechanical Factors
Source: Front Bioeng Biotechnol. 2021 Dec 8;9:765331. doi: 10.3389/fbioe.2021.765331 (PMC8692785; doi:10.3389/fbioe.2021.765331)
Supplement: Supplementary file 1 [file DataSheet1.docx]

Supplementary Material

# Ankle Joint Kinematics

Ankle motion was expressed in the OpenSim model using an anatomical description comprising dorsiflexion(+)/plantarflexion ($\theta_{q1}$) measured about a talocrural axis fixed in the tibia and supination(+)/pronation ($\theta_{q2}$) measured about a subtalar axis fixed in the talus (Fig. 1). Both $\theta_{q1}$ and $\theta_{q2}$ were defined to be initially zero in the nominal configuration of the foot as illustrated in Fig. 1.

Ankle rotations were also resolved in the ISB-recommended joint coordinate system (JCS) for the ankle joint complex [1] using a body-fixed zxy Euler angle rotation sequence relative to the frame embedded in the calcaneus. The calcaneus frame was assumed initially aligned with the tibia frame so that the nominal orientation of the foot as illustrated in Fig. 1 corresponded to non-zero JCS rotation angles. JCS rotations were expressed as dorsiflexion(+)/plantarflexion ($\theta_{dfx}$) measured about the Z-axis fixed in the tibia (which is identical to the talocrural anatomical axis and initially aligned with the z-axis fixed in the calcaneus), inversion(+)/eversion ($\theta_{inv}$) measured about the JCS floating axis (corresponding to the intermediate orientation of the x-axis fixed in the calcaneus, also called *line of nodes*), and internal(+)/external rotation ($\theta_{int}$) measured about the y-axis fixed in the calcaneus.


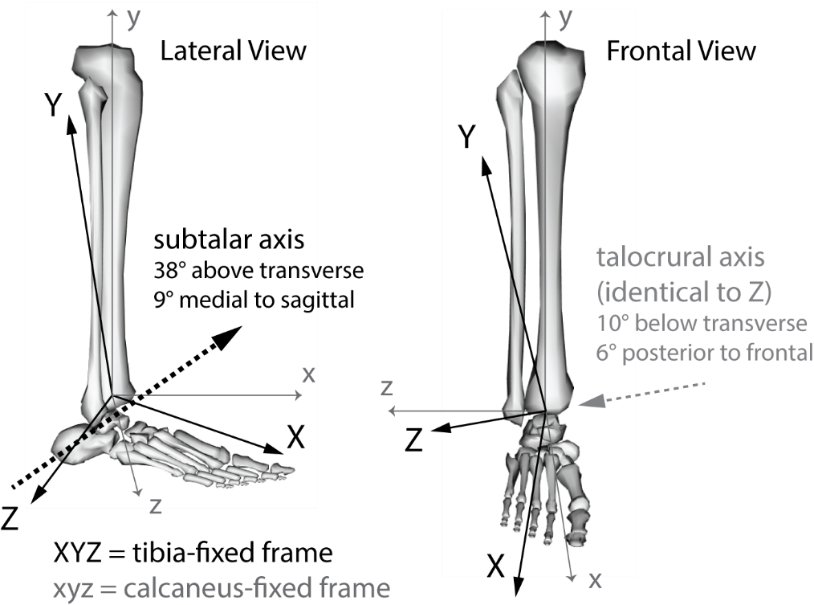


Figure S.1 - Reference axes used to resolve anatomic motion and joint coordinate system (JCS) mechanics of the ankle.

Anatomical rotation angles $\theta_{q1}$ and $\theta_{q2}$ as well as angular velocities $\dot{\theta}_{q1}$ and $\dot{\theta}_{q2}$ were used directly by the OpenSim model and reported as outcome metrics from the sprain simulation. JCS angles $\theta_{dfx}$, $\theta_{inv}$, $\theta_{int}$ were computed by constructing the body-fixed frames illustrated in Fig. 1 during each increment of the simulated sprain motion (please see [1] for details of axis definitions). JCS angles were then given by the following expressions,

$\boldsymbol{L=}\boldsymbol{j\times K}/\left\| \boldsymbol{j\times K} \right\|$ (1)

$\boldsymbol{\theta}_{\boldsymbol{dfx}}\boldsymbol{=}\boldsymbol{sin}^{\boldsymbol{-1}} \left( \boldsymbol{L\cdot J} \right)$ (2)

$\boldsymbol{\theta}_{\boldsymbol{inv}}\boldsymbol{=}\boldsymbol{sin}^{\boldsymbol{-1}} \left( \boldsymbol{K\cdot j} \right)$ (3)

$\boldsymbol{\theta}_{\boldsymbol{int}}\boldsymbol{=}\boldsymbol{sin}^{\boldsymbol{-1}} \left( \boldsymbol{L\cdot k} \right)$ (4)

where $L$ is a unit vector in the direction of the JCS floating axis (i.e., *line of nodes*), $ijk$ are unit vectors defining the axes of the calcaneus body-fixed frame, and $IJK$ are unit vectors defining the axes of the tibia body-fixed frame. All vectors are assumed to be expressed in components along the unit vectors of the global/ground frame.

JCS angular velocities were computed as follows. The total angular velocity vector of the calcaneus, expressed in terms of components along the unit vectors of the rotated calcaneus frame, may be defined either in terms of anatomical or JCS angular velocity values. In terms of anatomical angular velocity values, the total angular velocity vector is,

$\boldsymbol{\omega}_{\boldsymbol{calcn}}\boldsymbol{=}\boldsymbol{R}_{\boldsymbol{2}}\left( \boldsymbol{\theta}_{\boldsymbol{q}\boldsymbol{2}} \right) {\dot{\boldsymbol{\theta}}}_{\boldsymbol{q}\boldsymbol{1}}\left\{ \begin{matrix} \boldsymbol{tc}_{\boldsymbol{x}} \\ \boldsymbol{tc}_{\boldsymbol{y}} \\ \boldsymbol{tc}_{\boldsymbol{z}} \end{matrix} \right\}\boldsymbol{+}{\dot{\boldsymbol{\theta}}}_{\boldsymbol{q}\boldsymbol{2}}\left\{ \begin{matrix} \boldsymbol{st}_{\boldsymbol{x}} \\ \boldsymbol{st}_{\boldsymbol{y}} \\ \boldsymbol{st}_{\boldsymbol{z}} \end{matrix} \right\}$ (5)

where $R_{2}\left( \theta_{q2} \right)$ is a 3×3 orthonormal rotation matrix corresponding to the helical axis rotation around the subtalar axis, $\left\{ tc \right\}$ is a unit vector in the direction of the talocrural axis, and $\left\{ st \right\}$ is a unit vector in the direction of the subtalar axis. The $\left\{ tc \right\}$ vector can be thought to be expressed in terms of components along the unit vectors of either the tibia frame or the talus frame since it represents a hinge fixed in both the tibia and the talus and its components in either frame are invariant under rotation around itself. The $R_{2}\left( \theta_{q2} \right)$ term in equation (5) simply transforms the portion of angular velocity defined along $\left\{ tc \right\}$ so it is expressed in components along the unit vectors of the rotated calcaneus frame, which has also rotated around $\left\{ st \right\}$ to attain its final pose in any increment of motion.

Alternatively, in terms of JCS angular velocity values, $\omega_{calcn}$ may be written,

$\boldsymbol{\omega}_{\boldsymbol{calcn}}\boldsymbol{=}\boldsymbol{R}_{\boldsymbol{y}}\left( \boldsymbol{\theta}_{\boldsymbol{int}} \right) \boldsymbol{R}_{\boldsymbol{x}}\left( \boldsymbol{\theta}_{\boldsymbol{inv}} \right) \left\{ \begin{matrix} \boldsymbol{0} \\ \boldsymbol{0} \\ {\dot{\boldsymbol{\theta}}}_{\boldsymbol{dfx}} \end{matrix} \right\}\boldsymbol{+}\boldsymbol{R}_{\boldsymbol{y}}\left( \boldsymbol{\theta}_{\boldsymbol{int}} \right) \left\{ \begin{matrix} {\dot{\boldsymbol{\theta}}}_{\boldsymbol{inv}} \\ \boldsymbol{0} \\ \boldsymbol{0} \end{matrix} \right\}\boldsymbol{+}\left\{ \begin{matrix} \boldsymbol{0} \\ {\dot{\boldsymbol{\theta}}}_{\boldsymbol{int}} \\ \boldsymbol{0} \end{matrix} \right\}$ (6)

where rotations $R_{x}$, and $R_{y}$ are body-fixed rotation matrices given by,

$\boldsymbol{R}_{\boldsymbol{x}}\left( \boldsymbol{\theta}_{\boldsymbol{inv}} \right)\boldsymbol{=}\left[ \begin{matrix} \boldsymbol{1} & \cos\left( \boldsymbol{\theta}_{\boldsymbol{inv}} \right) & \sin\left( \boldsymbol{\theta}_{\boldsymbol{inv}} \right) \\ \boldsymbol{0} & \boldsymbol{-}\sin\left( \boldsymbol{\theta}_{\boldsymbol{inv}} \right) & \cos\left( \boldsymbol{\theta}_{\boldsymbol{inv}} \right) \\ \boldsymbol{0} & \boldsymbol{0} & \boldsymbol{0} \end{matrix} \right]$ ***(7)***

$\boldsymbol{R}_{\boldsymbol{y}}\left( \boldsymbol{\theta}_{\boldsymbol{int}} \right)\boldsymbol{=}\left[ \begin{matrix} \cos\left( \boldsymbol{\theta}_{\boldsymbol{int}} \right) & \boldsymbol{0} & \boldsymbol{-}\sin\left( \boldsymbol{\theta}_{\boldsymbol{int}} \right) \\ \boldsymbol{0} & \boldsymbol{1} & \boldsymbol{0} \\ \sin\left( \boldsymbol{\theta}_{\boldsymbol{int}} \right) & \boldsymbol{0} & \cos\left( \boldsymbol{\theta}_{\boldsymbol{int}} \right) \end{matrix} \right]$ ***(8)***

The right-hand side of (5) is completely known, so $\omega_{calcn}$ can be computed directly from the simulation outcome metrics. Substitution of (7) and (8) into (6) then reduces to,

$\left\{ \begin{matrix} \boldsymbol{\omega}_{\boldsymbol{calcn, x}} \\ \boldsymbol{\omega}_{\boldsymbol{calcn, y}} \\ \boldsymbol{\omega}_{\boldsymbol{calcn, z}} \end{matrix} \right\}\boldsymbol{=}\left\{ \begin{matrix} {\dot{\boldsymbol{\theta}}}_{\boldsymbol{inv}}\cos\left( \boldsymbol{\theta}_{\boldsymbol{int}} \right)\boldsymbol{-}{\dot{\boldsymbol{\theta}}}_{\boldsymbol{dfx}}\cos\left( \boldsymbol{\theta}_{\boldsymbol{inv}} \right)\sin\left( \boldsymbol{\theta}_{\boldsymbol{int}} \right) \\ {\dot{\boldsymbol{\theta}}}_{\boldsymbol{int}}\boldsymbol{+}{\dot{\boldsymbol{\theta}}}_{\boldsymbol{dfx}}\sin\left( \boldsymbol{\theta}_{\boldsymbol{inv}} \right) \\ {\dot{\boldsymbol{\theta}}}_{\boldsymbol{inv}}\sin\left( \boldsymbol{\theta}_{\boldsymbol{int}} \right)\boldsymbol{+}{\dot{\boldsymbol{\theta}}}_{\boldsymbol{dfx}}\cos\left( \boldsymbol{\theta}_{\boldsymbol{inv}} \right)\cos\left( \boldsymbol{\theta}_{\boldsymbol{int}} \right) \end{matrix} \right\}$ ***(9)***

Therefore, (5) and (9) can be used to find $\dot{\theta}_{dfx}$ and $\dot{\theta}_{inv}$ by solving a system of two equations in two unknowns,

$\left\{ \begin{matrix} \boldsymbol{\omega}_{\boldsymbol{calcn, x}} \\ \boldsymbol{\omega}_{\boldsymbol{calcn, z}} \end{matrix} \right\}\boldsymbol{=}\left[ \begin{matrix} \boldsymbol{-}\cos\left( \boldsymbol{\theta}_{\boldsymbol{inv}} \right)\sin\left( \boldsymbol{\theta}_{\boldsymbol{int}} \right) & \cos\left( \boldsymbol{\theta}_{\boldsymbol{int}} \right) \\ \cos\left( \boldsymbol{\theta}_{\boldsymbol{inv}} \right)\cos\left( \boldsymbol{\theta}_{\boldsymbol{int}} \right) & \sin\left( \boldsymbol{\theta}_{\boldsymbol{int}} \right) \end{matrix} \right]\left\{ \begin{matrix} {\dot{\boldsymbol{\theta}}}_{\boldsymbol{dfx}} \\ {\dot{\boldsymbol{\theta}}}_{\boldsymbol{inv}} \end{matrix} \right\}$ ***(10)***

and simple substitution of the result into the second row of (9) then gives $\dot{\theta}_{int}$.

# Supplementary Figures and Tables

Figure S.6 - Probabilistic sensitivity factors of subtalar peak supination angle in response to changes in input parameter mean and variance, for six Monte Carlo (MC) simulations. Sensitivity factors are normalized by parameter variance and response probability level to enable relative comparison across parameters.

| **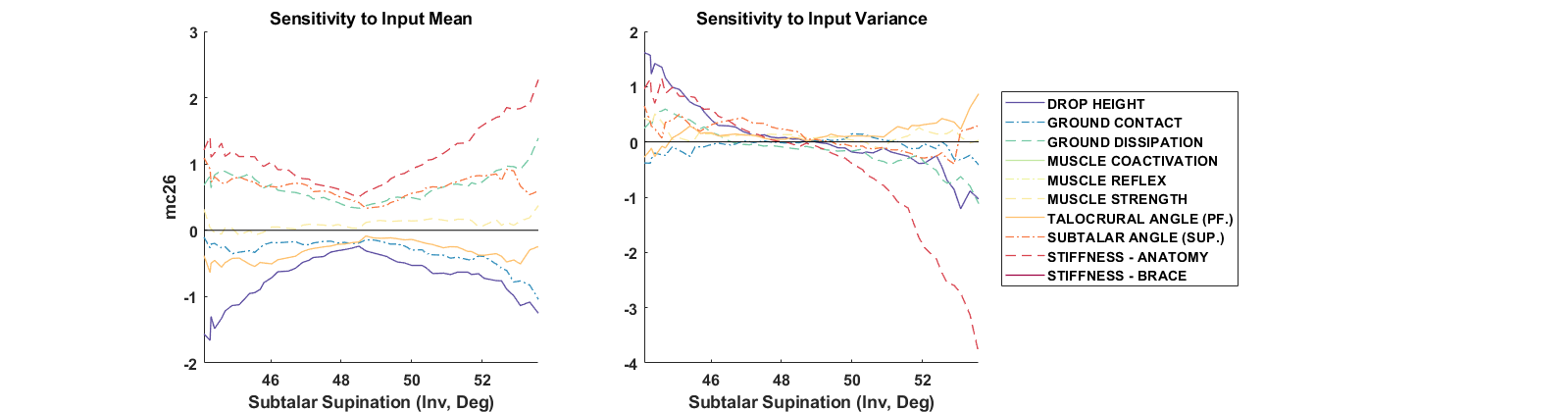** | |
| --- | --- |
| **MC Study 1: C= 0%, B=0%, R=0 (Level)** | **MC Study 2: C= 0%, B=0%, R=0 (Incline)** |
| **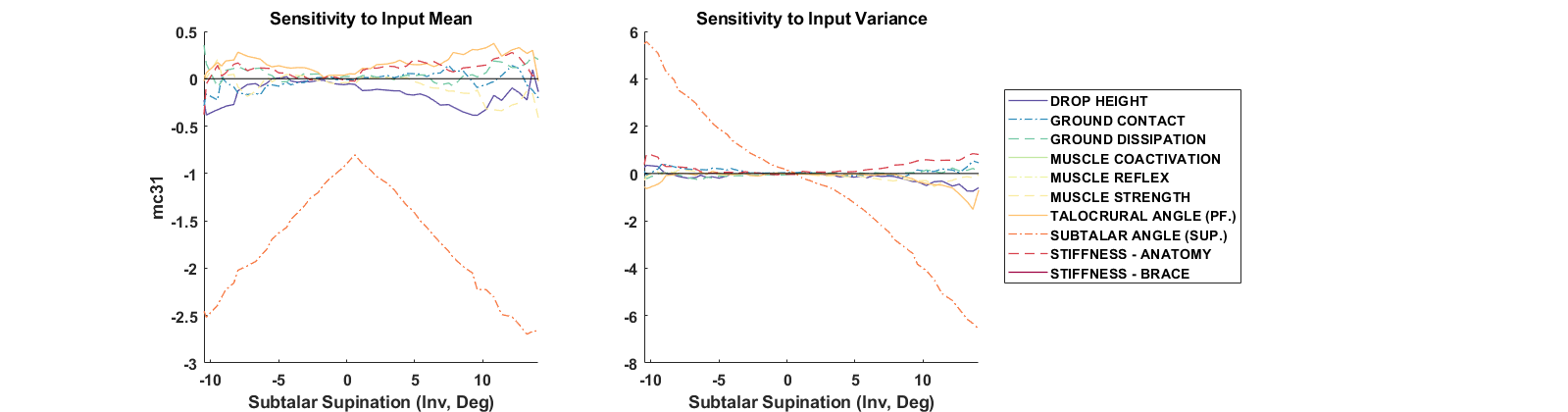** | **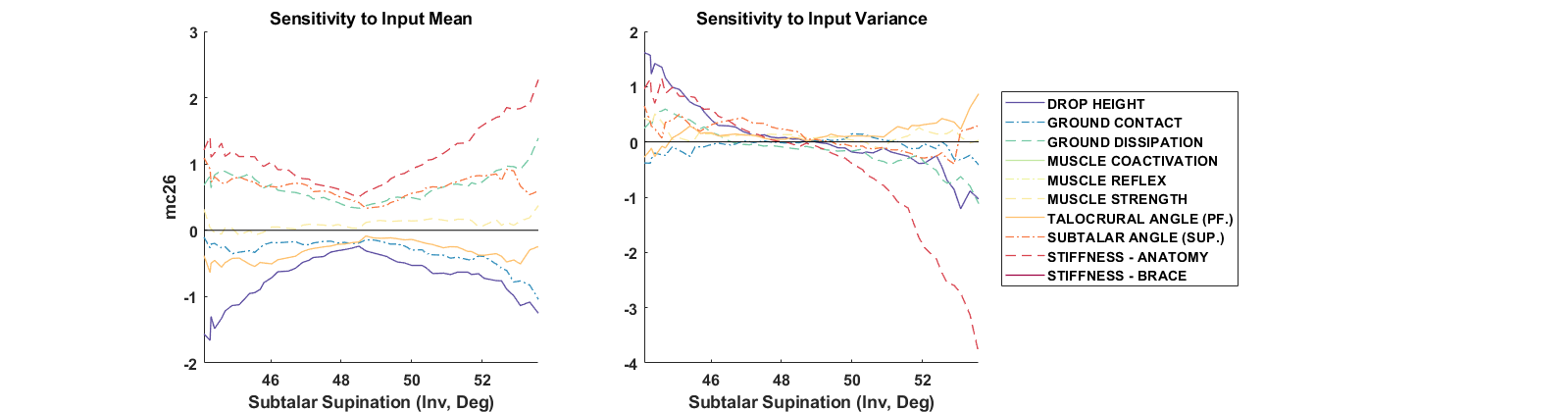** |
| **MC Study 3: C= 0%, B=0%, R=10 (Incline)** | **MC Study 4: C= 60%, B=0%, R=0 (Incline)** |
| **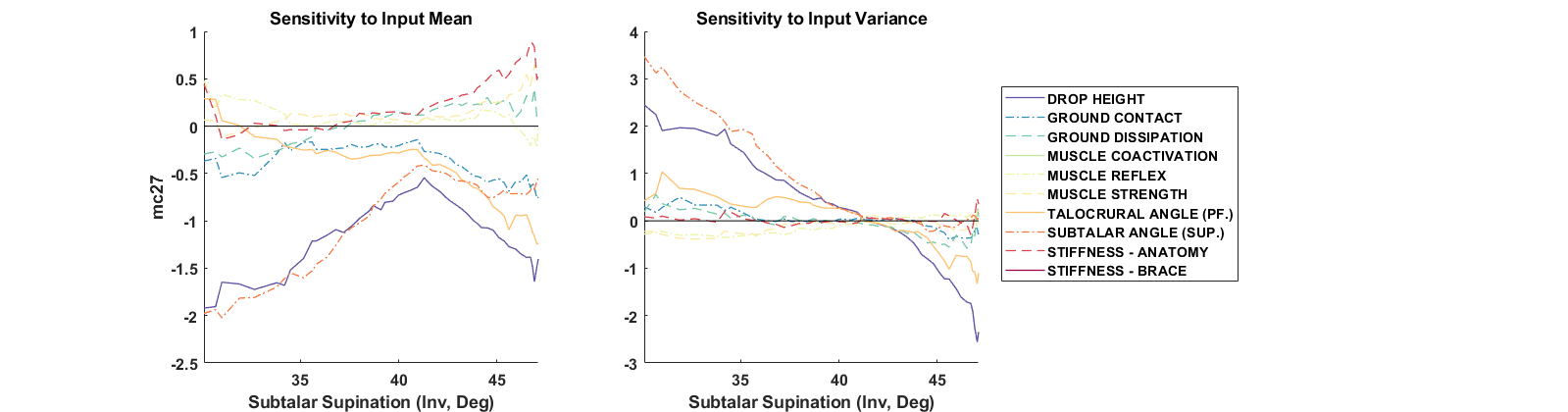** | **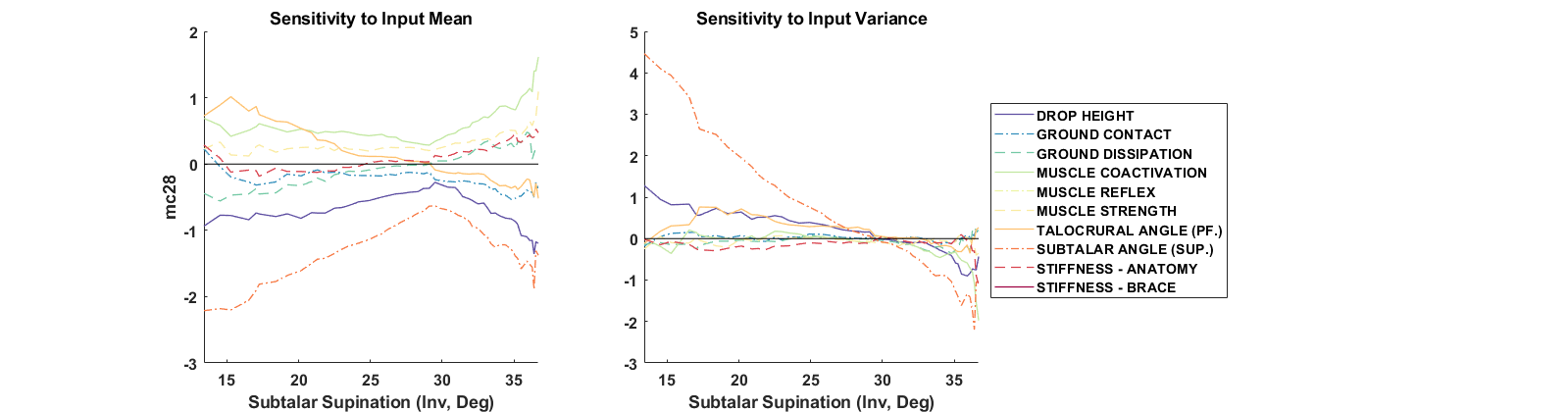** |
| **MC Study 5: C= 0%, B=240%, R=0** **(Incline)** | **MC Study 6: C= 20%, B=150%, R=5 (Incline)** |
| **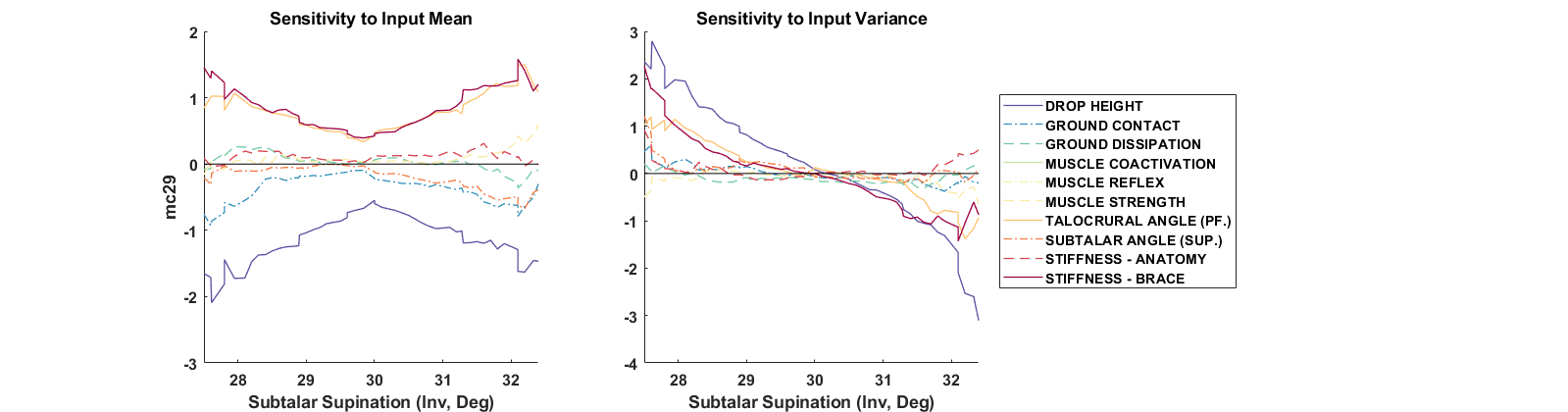** | **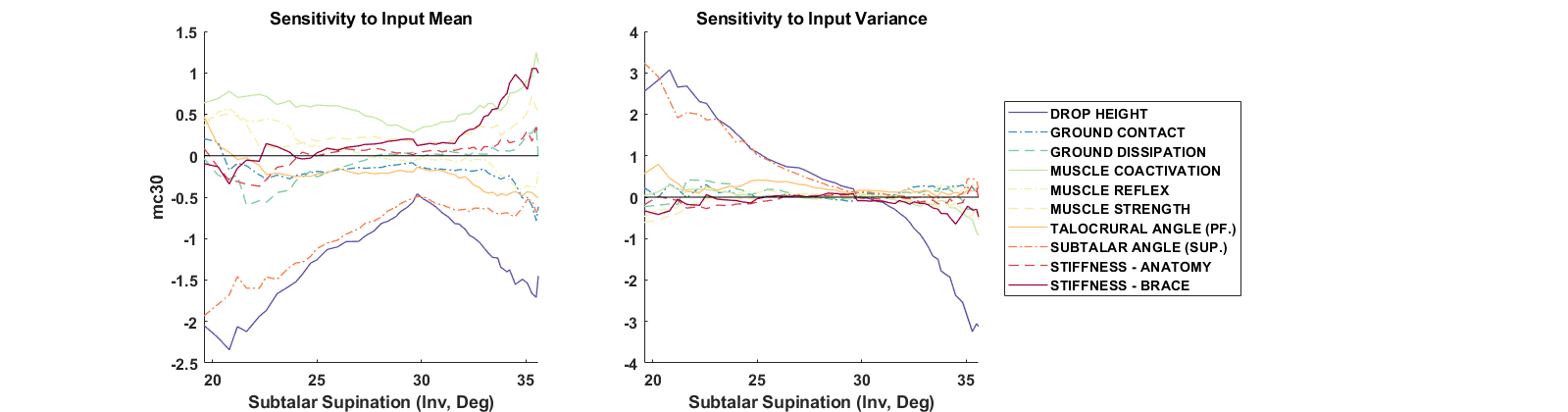** |
